# Durlobactam in the Treatment of Multidrug-Resistant *Acinetobacter baumannii* Infections: A Systematic Review

**DOI:** 10.3390/jcm11123258

**Published:** 2022-06-07

**Authors:** Guido Granata, Fabrizio Taglietti, Francesco Schiavone, Nicola Petrosillo

**Affiliations:** 1 Clinical and Research Department for Infectious Diseases, National Institute for Infectious Diseases L. Spallanzani, IRCCS, 00149 Rome, Italy; guido.granata@inmi.it (G.G.); fabrizio.taglietti@inmi.it (F.T.); 2 Divers and Raiders Group Command “Teseo Tesei” COMSUBIN, Medical Service, Italian Navy, 19025 Portovenere, Italy; francesco.schiavone.san@gmail.com; 3 Head, Infection Prevention and Control–Infectious Disease Service, Foundation University Hospital Campus Bio-Medico, 00128 Rome, Italy

**Keywords:** durlobactam, *A. baumannii*, systematic review, sulbactam, multidrug resistance

## Abstract

*A. baumannii* is a frequent cause of difficult-to-treat healthcare-associated infections. The use of a novel beta-lactamase inhibitor, durlobactam, has been proposed against multidrug-resistant *A. baumannii*. A systematic review of studies assessing the efficacy and safety of durlobactam in the treatment of multidrug-resistant *A. baumannii* infections was carried out. The study protocol was pre-registered on PROSPERO (CRD42022311723). Published articles on durlobactam were identified through computerized literature searches with the search terms “durlobactam” and “ETX2514” using PubMed. PubMed was searched until 15 February 2022. Articles providing data on the main characteristics of durlobactam and on the efficacy and safety of durlobactam in the treatment of *A. baumannii* infections were included in this systematic review. Attempt was made to obtain information about unpublished studies. English language restriction was applied. The risk of bias in the included studies was not assessed. Both quantitative and qualitative information were summarized by means of textual descriptions. Thirty studies on durlobactam were identified, published from June 2017 to November 2020. Sixteen studies met the inclusion criteria. Durlobactam is effective against *A. baumannii* when used in combination with sulbactam. Future clinical trials are needed to confirm the possibility to treat infections caused by multidrug-resistant *A. baumannii* with this combination.

## 1. Introduction

Worldwide, the rise of multidrug-resistant (MDR) bacteria is an increasing threat to human health [1,2]. Among the most worrisome MDR bacteria, the World Health Organization (WHO) recently recognized *Acinetobacter baumannii* as a critical pathogen, frequently causing healthcare-associated infections (www.who.int accessed on 1 May 2022).

*A. baumannii* is a ubiquitous, non-fermenting, rod-shaped Gram-negative coccobacillus. It can be considered an opportunistic pathogen, because predominantly affects immune-compromised and critically ill patients [3,4]. In this particularly frail population, *A. baumannii* can cause ventilator-associated pneumonia and bloodstream infections, with an overall reported mortality up to 40% [5,6]. *A. baumannii* possesses the pernicious, innate ability to evade the commonly used antibiotic therapy. 

Generally, *A. baumannii* resistance mechanisms of intrinsic and acquired antibiotic resistance are categorized into three groups. First, resistance can be achieved by increasing efflux of the antibiotic from the bacteria and thus preventing access to the target, i.e., overexpression of drug efflux pumps. Second, *A. baumannii* can protect its antibiotic target through genetic mutations or post-translational modifications, i.e., mutations in antibiotic binding targets via genetic insertion sequences. Third, antibiotics can be directly inactivated by hydrolysis, i.e., enzymatic inactivation by beta-lactamases. To consider, multiple different resistance mechanisms are often present at the same time in *A. baumannii* [7,8,9].

As for other bacteria, MDR *A. baumannii* can be defined as an organism showing acquired non-susceptibility to at least one agent in three or more antimicrobial categories [10].

Unfortunately, thus far, the clinical trials on the treatment of MDR *A. baumannii* infections have not provided with conclusive evidence in favor of one antibiotic over another. Antibiotic selection relies upon interpretation of in vitro efficacy, host factors, pharmacokinetic (PK) and pharmacodynamic (PD) profiles [11]. Generally, an antimicrobial combination approach is chosen to overcome the multiple mechanisms of resistance and suppress further resistance. However, the real clinical benefit of combination treatments for infections due to MDR *A. baumannii* remains unclear.

Moreover, the quiver of antimicrobials retaining in vitro activity against MDR *A. baumannii* strains is generally restricted to few classes, i.e., aminoglycosides, polymyxins, and some tetracyclines. These antimicrobials share important limitations, such as site-specific pharmacokinetics, emergence of resistance and toxicity.

Recently, studies proposed to use a novel compound, durlobactam, against MDR *A. baumannii*. Durlobactam is a novel beta-lactamase inhibitor, effective against *A. baumannii* and MDR *A. baumannii* when used in combination with another beta-lactamase inhibitor with intrinsic antibacterial activity against *A. baumannii*, i.e., sulbactam [12]. 

We performed a systematic review of the literature with the main aim to summarize available evidence supporting durlobactam use in the treatment of MDR *A. baumannii* infections. 

## 2. Materials and Methods

### 2.1. Search Strategy and Article Identification

The study protocol was pre-registered on PROSPERO (CRD42022311723) (Tables S1 and S2). Published articles (from June 2017 to November 2020) assessing the main characteristics of durlobactam and the efficacy and safety of durlobactam in the treatment of *A. baumannii* infections were identified through computerized literature searches using MEDLINE (National Library of Medicine Bethesda MD) and by reviewing the references of retrieved articles. PubMed was searched until 15 February 2022. Combinations of the following search terms were applied: ((durlobactam) OR (ETX2514)). Attempt was made to obtain information about unpublished studies. English language restriction was applied. Studies published only in abstract form, correction articles, reviews, case-reports, editorials, guidance articles or guidelines and clinical trial protocols were excluded from further assessment. Reviewed articles were maintained in a master log and any reason for exclusion from analysis was documented in the rejected log.

### 2.2. Eligibility Criteria

Studies of any design which reported data on durlobactam were eligible for inclusion in this systematic review.

### 2.3. Study Selection and Data Extraction

Eligibility assessment and extraction of data were performed independently by two investigators. Each investigator was blinded to the other investigator’s data extraction. In case of disagreement between the two reviewers, a third reviewer was consulted. Data from each study were verified for consistency and accuracy, and then entered into a computerized database. Abstracted information included: author, year of publication, country in which the study was conducted; study design, start and end date of study, health-care setting, sample size; criteria for the diagnosis of bacterial infection, if applicable; proportion of animals or patients receiving antibiotics, if applicable; minimum inhibitory concentration; pharmacokinetics and pharmacodynamics data; animals or patients’ outcome data. For the syntheses, studies were grouped in two groups. Group I: in vitro studies on general characteristics and pharmacokinetic of durlobactam and on the activity of durlobactam against *A. baumannii* isolates. Group II: clinical studies on the efficacy of durlobactam against infections due to *A. baumannii*.

### 2.4. Data Synthesis

Both quantitative and qualitative information were summarized by means of textual descriptions.

### 2.5. Assessment of Bias

A formal assessment for risk of bias was deemed to have limited utility given the lack of an appropriate assessment tool. Although a risk-of-bias tool has been developed for systematic reviews, many aspects of the tool are not directly relevant to our research question.

## 3. Results

### 3.1. Studies Description

**Figure 1:**
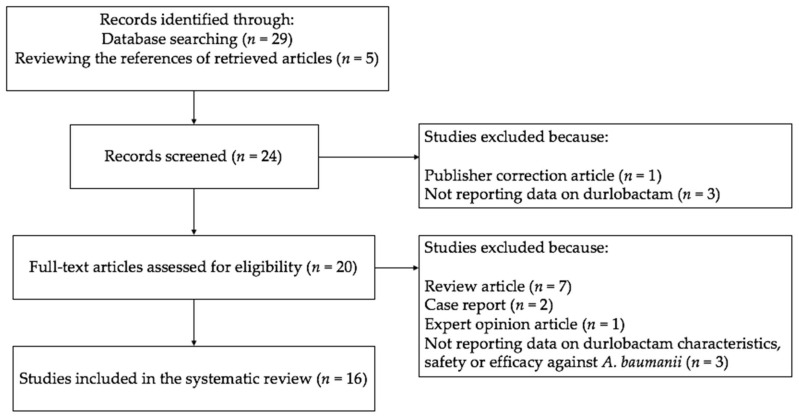
Flowchart depicting the selection process of studies included in the systematic review.

Figure 1 shows the selection process of studies included in the systematic review. Through a PubMed search with the search terms “durlobactam” and “ETX2514”, we identified 29 studies published from June 2017 to November 2020. Among the 29, five studies that represented review articles, four studies that did not report data on durlobactam, one study representing a “correction article”, one representing an “expert opinion article”, and two representing case reports were excluded. Of the 16 studies included in this systematic review (Figure 1) [13,14,15,16,17,18,19,20,21,22,23,24,25,26,27,28], six were clinical trials: four phase I trials [16,17,19,27], one phase II clinical trial [18] and one ongoing phase III clinical trial [28].

Of the 16 studies included in the systematic review, in vitro studies and phase I and phase II studies produce data on general characteristics, pharmacokinetic and pharmacodynamic of durlobactam; seven studies assess the in vitro activity of durlobactam against *A. baumannii* from patients; one was an ongoing phase III trial study on the efficacy of durlobactam against *A. baumannii*. The included studies were separated in two different tables with a summary description of their characteristics: Table 1, in vitro studies and Table 2, Phase I, phase II or phase III studies. 

### 3.2. Durlobactam Characteristics

#### 3.2.1. Durlobactam Molecular Structure and Pharmacodynamics

**Figure 2:**
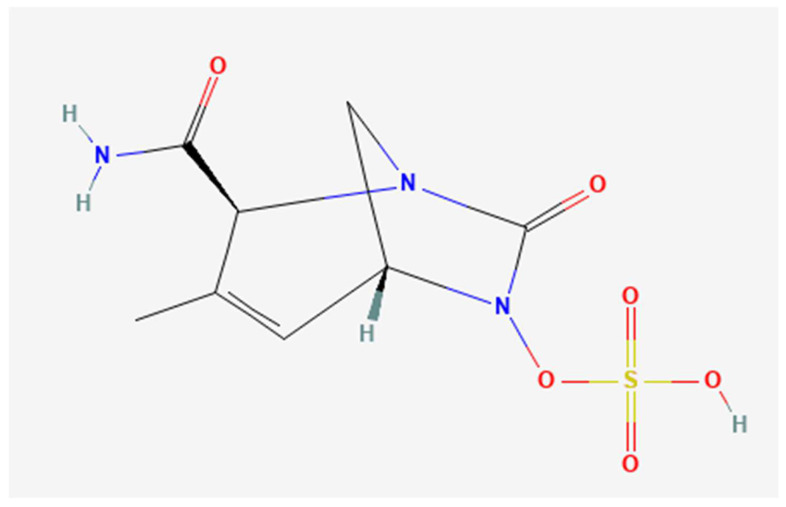
Durlobactam chemical structure.

**Figure 3:**
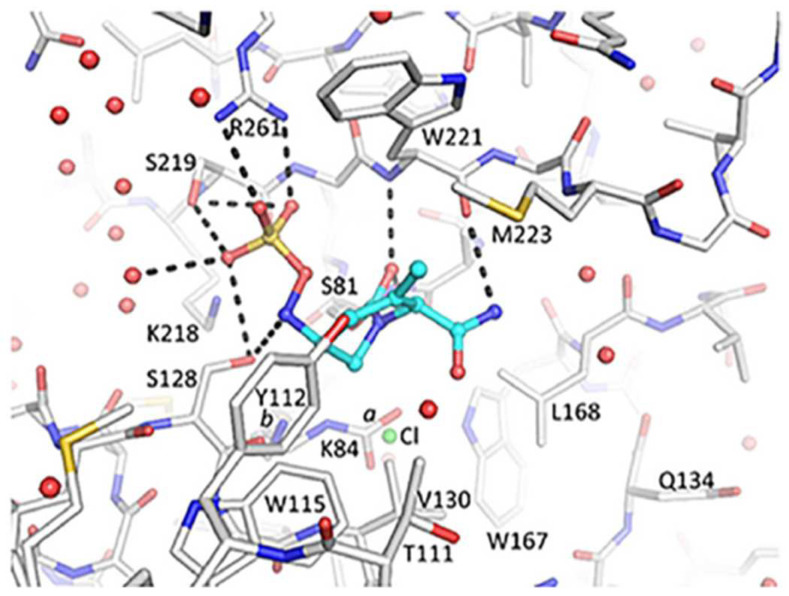
X-ray crystal structure of durlobactam in covalent complex with OXA-24/40 at 2.0 Å resolution (PDB: 6MPQ). Reproduced from Barnes MD et al. (2019) with permission from the authors.

Durlobactam, formerly known as ETX2514, is a novel beta-lactamase inhibitor belonging to the diazabicyclooctanone, boronic acid and pyridine-2-carboxylic acid classes {[(2S,5R)-2-carbamoyl-3-methyl-7-oxo-1,6-diazabicyclo [3.2.1]oct-3-en-6-yl] hydrogen sulfate} [13]. Durlobactam molecular structure differs from other beta-lactamases for its endocyclic double bond and its methyl substituent (Figure 2 and Figure 3). Durlobactam is a polar compound, therefore it is able to penetrate into Gram-negative cells through outer membrane porins, i.e., OmpA [26]. Once into the bacterial cell, durlobactam is carbamoylated on its active, nucleophile serine site. This covalent bond is reversible because the sulfated amine is able to recyclize onto the carbamate [15]. These molecular characteristics confer a favorable binding kinetic and extend durlobactam inhibitory effect to a broad range of beta-lactamases, including Amber class A, C and D beta-lactamases [15]. Durlobactam is able to bind penicillin-binding proteins in a rapid and reversible manner, resulting in an efficient and long-lasting inhibition [15].

Durlobactam potently inhibits clinically relevant Amber class A, C, and D beta-lactamases. More precisely, durlobactam recyclizes and dissociates intact from beta-lactams belonging to class A and C, including AmpC, CTX-M-15, P99, SHV-5, and TEM-1 [15]. Even if durlobactam does not completely dissociates intact from other classes A and D beta-lactams, such as KPC-2, OXA-10, OXA-23, OXA-24, OXA-48, it retains in vitro inhibition of these beta-lactamases. Importantly, durlobactam does not inhibits class B metallo-beta-lactamases [15].

However, the sole durlobactam has minimal antibacterial activity against *A. baumannii*, mainly due to its ability to bind penicillin-binding proteins (PBP) 2 [13,20,21,22,23,24,25].

#### 3.2.2. Durlobactam Pharmacokinetics

Durlobactam is suitable for intravenous administration, with stability for more than 6 h at room temperature and a water solubility higher than 200 mg/mL [13].

As others beta-lactam inhibitors, durlobactam shows a linear pharmacokinetics, allowing prompt dose adjustments when required. The durlobactam estimated volume of distribution at steady state can be described by a two-compartment model and it is influenced by patient body weight [13,16,17,18,19]. The systemic clearance of durlobactam does not change after single and multiple doses (0.25–2.0 g) [16,17,18,19]. The infusion of durlobactam, alone or in combination with sulbactam, is generally safe and well tolerated [16,17,18,19,27].

The pharmacokinetics and pharmacodynamics of durlobactam in combination with sulbactam have been studied in a phase I trial enrolling 30 healthy adults (ClinicalTrials registration no. NCT03303924) [17].

In this trial, 1 g of durlobactam with 1 g of sulbactam were infused as 3 h intravenous infusion every 6 h, for three consecutive doses. At these regimens, the durlobactam/sulbactam combination was safe and generally well tolerated. In this study the concentration of durlobactam and sulbactam were determined in plasma and epithelial lining fluid. Generally, the total plasma concentrations of each agent resulted higher than those observed in the epithelial lining fluid. The times of the observed maximum plasma concentration (Tmax) were similar for durlobactam and sulbactam, with arithmetic means of 2.62 and 2.56 h, respectively. The means of the minimum plasma concentrations (C min) of durlobactam at 6 h after the first, second, and third infusions were 5.71 ± 1.77, 6.63 ± 1.97, and 5.79 ± 2.08 mg/L, respectively [17].

Of note, following the third infusion, durlobactam showed values of area under the concentration-time curves from 0 to 6 h (AUC0-6) of 109.05 ± 23.44 mg/h/L, the half-life of 1.40 ± 0.18 h and a volume of distribution at steady state of 16.7 ± 3.0 L [17].

Moreover, regarding durlobactam distribution to the epithelial fluid, the AUC0–6 based on mean epithelial lining fluid concentrations resulted 40.1 mg/h/L. The ratio of epithelial lining fluid to total plasma durlobactam concentrations based on the mean AUC0–6 values was 0.37. These results support the use of the durlobactam/sulbactam combination in the treatment of lower respiratory tract bacterial infections [17].

A subsequent, phase II clinical trial performed on 80 patients with complicated urinary tract infection confirmed these findings. In this study, patients were randomized to receive imipenem plus the combination of durlobactam/sulbactam at a regimen of 1 plus 1 g, infused over 3 h every 6 h for 7 days (n: 53), or imipenem plus placebo (n: 27) [18]. The durlobactam/sulbactam combination was generally well tolerated, with 37.7% patients reporting drug-related moderate adverse events such as headache, diarrhea, nausea, and phlebitis. One patient had self-limiting urticaria on day 3 of administration. In one patient, renal function decrease was observed on day 3 of administration. In this phase II trial, durlobactam and sulbactam mean elimination half-lives were of 2.2 and 1.6 h, respectively. The mean steady-state clearance and volume of distribution of durlobactam were 10.3 L/h and 31.6 L, respectively. These values were similar to the mean clearance and volume of distribution estimates for sulbactam (13.4 L/h and 36.0 L, respectively) [18]. On these findings, the authors proposed a durlobactam/sulbactam dose of 1 g of each component to be administered every 6 h via a 3 h infusion to achieve the optimal concentrations [18].

To consider, although the urinary excretion of durlobactam over 48 h is only 66% of the systemic clearance, durlobactam exposure doubled in patients with renal impairment with creatinine clearance lower than 30 mL/min/1.73 m^2^ [21]. Therefore, changes in durlobactam pharmacokinetics should be considered in patients with severely compromised renal function and hemodialysis should be considered as a factor that can remove nearly half a dose of durlobactam [19].

### 3.3. In Vitro Studies on the Activity of Durlobactam/Sulbactam against A. baumannii

#### Durlobactam and Sulbactam Synergistic Bactericidal Activity

The sole durlobactam possesses minimal antibacterial activity against *A. baumannii*, mainly due to durlobactam ability to bind *A. baumannii* PBPs [13].

Durlobactam showed a synergistic bactericidal activity in combination with beta-lactam antibiotics [14,20,21,22,23,24,25]. Durlobactam improves the activity of several beta-lactam partners tested against *A. baumannii*, but the most potent combination has been observed with another beta-lactamase inhibitor, sulbactam [14,20,21,22,23,24,25].

Sulbactam is a well-known beta-lactamase inhibitor which also possesses antibacterial activity against *A. baumannii*, due to its selective binding to the PBP 1, 2, and 3 [14,20,21,22,23,24,25].

However, in the last decades, MDR *A. baumannii* strains emerged with a reduced expression of PBP2 and produced beta-lactamases able to degrade sulbactam, such as TEM-1, ADC-30, AmpC and a number of OXAs. Unfortunately, these resistance mechanisms narrowed the clinical utility of the sole sulbactam in MDR *A. baumannii* infections [23,25].

Therefore, considering that the sole durlobactam does not have significant activity against *A. baumannii*, its use in combination with sulbactam has been proposed. Exploiting inhibition of beta-lactamases by durlobactam, sulbactam is free to exert its intrinsic *A. baumannii* bactericidal activity by binding PBPs [23,25].

Sulbactam alone is weakly active against MDR *A. baumannii* clinical isolates, but when combined with durlobactam, minimal inhibitory concentration (MIC) 90 drops to 16-fold, to 4 mg/L [14,20,21,22,23,24,25]. The durlobactam/sulbactam combination showed activity against Ambler class A, C and D beta-lactamases, possessing a potential utility for the treatment of infections due to MDR *A. baumannii* [14,20,21,22,23,24,25].

To date, the frequency of *A. baumannii* spontaneous resistance to the durlobactam/sulbactam combination seems low, even if resistance has been recently reported due to the metallo-beta-lactamase NDM−1 or PBP3 substitutions [20].

In a recent study, the in vitro antibacterial activities of the durlobactam/sulbactam combination were assessed by broth microdilution against 1722 *A. baumannii* clinical isolates, collected across the five continents in 2016 and 2017 [14]. Over 50% of these samples resulted resistant to carbapenems. Against this strain collection, durlobactam/sulbactam showed MIC_50_ and MIC_90_ values of 1 and 2 g/mL, respectively. These MICs were lower than that observed for the sole sulbactam, with a MIC_50_ of 8 g/mL and a MIC_90_ of 64 g/mL. This level of activity was found to be consistent across regions, sources of infection, and subsets of resistance phenotypes, including MDR isolates. Of note, in this study colistin was the only traditional antimicrobial with activity similar to durlobactam/sulbactam. Moreover, genome sequencing of the 39 specimens (2.3%) with a durlobactam/sulbactam MIC of 4 g/mL confirmed that these strains encoded either the metallo-beta-lactamase NDM-1, not inhibited by durlobactam, or single amino acid substitutions near the active site of PBP3, i.e., the primary target of sulbactam [14].

Similar results were reported in a study on 246 patients infected by *A. baumannii* collected between 2012 and 2016 from 94 hospitals in 37 different countries. Antimicrobial susceptibility testing was performed by broth microdilution. The observed durlobactam/sulbactam MIC_50_ and MIC_90_ values were 0.25 and 0.5 mg/L, respectively. Sulbactam MIC was lowered 16-fold to 64-fold by the addition of durlobactam. Conversely, the addition of imipenem to the durlobactam/sulbactam combination did not improve activity, with only nine isolates showing higher MIC. These samples encoded the metallo-beta-lactamase NDM-1 [21].

In an in vitro study on 982 samples of *A. baumannii* infection in China between 2016 and 2018, the observed MIC_90_ of durlobactam/sulbactam was 2 mg/L [22]. In this study, 84.6% of the collected strains was imipenem resistant. The authors of this study propose a concentration of 4 mg/L as a reasonable preliminary cut-off for evaluation of durlobactam/sulbactam susceptibility in *A. baumannii*.

Moreover, a recent study used whole-genome sequencing and molecular characterization to relate the presence of carbapenemase genes in 28 *A. baumannii* clinical isolates from India. The authors found that 93% of the collected samples expressed carbapenemases, including OXA-23, OXA-58 and NDM genes, with over a third expressing dual carbapenemase genes. The presence of these carbapenemase genes resulted in sulbactam resistance (MIC: 16–256 mg/L) in all of the studied isolates [23].

The authors then assessed the efficacy of durlobactam against these strains through in silico intermolecular interaction analysis. Several nonsynonymous single nucleotide polymorphisms were identified in PBP2 and PBP3 sequences, but minimal variations were recorded in the protein backbone dynamics in active-site motifs of wild-type and mutants, which correlated with negligible binding energy fluctuations for the PBP2-durlobactam complex. The authors suggested that the stable interaction profiles of durlobactam with carbapenemases can possibly restore sulbactam activity against both wild type and PBP mutant examinee [23].

Finally, a recent study evaluated the antimicrobial activity of durlobactam/sulbactam against a collection of 112 Brazilian MDR *A. baumannii* patients [24]. The in vitro activity of durlobactam/sulbactam was evaluated by the broth microdilution method using durlobactam at a fixed concentration of 4 mg/L. In this study, the samples presented a variety of beta-lactamases encoding genes, including several OXAs. Despite the high resistance rates to most antimicrobial agents tested, the authors reported in vitro activity of durlobactam/sulbactam, with MIC_90_ values of 4 mg/L [24].

### 3.4. In Vivo Studies on the Efficacy of Durlobactam/Sulbactam to Treat A. baumannii Infections

A study on the efficacy of durlobactam/sulbactam against MDR *A. baumannii* was performed in the animal model. In thigh and lung murine infection models, durlobactam/sulbactam showed a dose dependent reduction in *A. baumannii* counts. Bactericidal activity of durlobactam/sulbactam greater than one-log kill was achieved when sulbactam concentrations exceeded the combination MIC of 0.5 mg/L. In this study, no bactericidal activity was observed when sulbactam was administered alone, at the dosage of 15 mg/kg every three hours. The addition of durlobactam to sulbactam increased its activity in a dose-dependent manner [25].

Thus far, in human durlobactam/sulbactam combination has been studied in multiple phase I trials [16,17,19], one phase II trial [18], and in an ongoing phase III trial (NCT03894046) [28].

A phase II trial evaluated the tolerability and pharmacokinetic of durlobactam/sulbactam in patients with complicated urinary tract infections, including acute pyelonephritis. In this study all patients received background therapy with imipenem, in addition to either durlobactam/sulbactam or placebo. The microbiological intent-to-treat population were similar in the two groups, 36 (76.6%) and 17 (81.0%) patients in the durlobactam/sulbactam and in the placebo group, respectively [18].

Currently, a phase III trial, i.e., the ATTACK trial, is evaluating the efficacy and safety of the durlobactam/sulbactam combination in patients with bloodstream infections or hospital-associated bacterial pneumonia due to *A. baumannii-calcoaceticus complex* (NCT03894046) [28]. The first part of this multinational, pathogen-targeted, randomized, active-controlled, comparator-controlled trial compared two treatment arms: durlobactam/sulbactam (1 g/1 g qid) plus imipenem/cilastin (1 g/1 g qid) versus colistin (2.5 mg/kg bid) plus imipenem/cilastin (1 g/1 g qid). This trial concluded the patients’ recruitment, with a total of 207 enrolled patients, and recently an update on the study results has been released [28].

Approximately 95% of baseline examinees tested were carbapenem resistant. Durlobactam/sulbactam achieved statistical non-inferiority to colistin in the 28-day all-cause mortality efficacy endpoint. Mortality analyses favored durlobactam/sulbactam versus colistin in microbiologically modified intent-to-treat population. Durlobactam/sulbactam mortality was 19.0% compared to 32.3% in the colistin arm (treatment difference of −13.2%; 95% CI: −30.0, 3.5) [28].

At test of cure, there was a statistically significant difference in clinical response favoring durlobactam/sulbactam over colistin (61.9% versus 40.3%, respectively; 95% CI: 2.9, 40.3). Moreover, durlobactam/sulbactam met the primary safety objective of the trial, achieving statistically significant reduction in nephrotoxicity [28].

## 4. Discussion

Globally, severe infections due to MDR *A. baumannii* give rise to high mortality rates and remain a great challenge for clinicians [6,7].

Among the reasons to explain this increased mortality, there are the potential toxicity and the suboptimal pharmacokinetics and efficacy of the currently available therapeutic approaches [29,30].

During the last decades, polymyxins represented the most used antimicrobial options against MDR *A. baumannii*. Polymyxins have been considered as a “last resort” to fight MDR infections, often representing the only antimicrobial to achieve adequate serum levels and MICs. Therefore, the reports of colistin-resistant *A. baumannii* isolates raises concern, considering the further limitations of antimicrobial options and the high mortality rate associated with these infections [4].

Unfortunately, newer beta-lactams and beta-lactamase inhibitor combinations such as ceftolozane–tazobactam, ceftazidime–avibactam, imipenem–relebactam and meropenem–vaborbactam are clinically ineffective against *A. baumannii* [31].

Among the most recently developed antimicrobials, cefiderocol and eravacycline may be considered as salvage therapy against MDR *A. baumannii* [32,33]. However, cefiderocol and eravacycline recently showed disappointing clinical outcomes data against MDR *A. baumannii* [12,32,33,34]. Regarding cefiderocol, it showed in vitro activity against MDR *A. baumannii*, but its in vivo efficacy is variable [34].

In summary, the in vitro studies demonstrated that durlobactam and sulbactam permeates the *A. baumannii* outer membrane protein A (OmpA) [26]. After penetrating the bacterial cell, durlobactam recyclizes and dissociates intact from several beta-lactamases [15]. Against *A. baumannii* isolates, durlobactam/sulbactam showed MIC_50_ values ranging between 0.5 and 1 mg/mL and MIC_90_ values from 2 to 4 mg/mL [14,20,21,22].

The clinical studies demonstrated that durlobactam is safe and generally well tolerated when administered in combination with sulbactam [16,27]. Durlobactam showed a half-life of 1.40 ± 0.18 h and a mean steady-state clearance and volume of distribution of 10.3 L/h and 31.6 L, respectively [17,18]. The predominant clearance mechanism is through renal excretion [16]. Decreasing renal function increases peak plasma concentration and AUC in a generally linear manner and hemodialysis is effective at removing both durlobactam and sulbactam from plasma [19]. In an ongoing phase III clinical trial comparing durlobactam/sulbactam and colistin for the treatment of carbapenem-resistant *A. baumannii* infection, durlobactam/sulbactam met the primary efficacy endpoint of all-cause mortality at 28 days (durlobactam/sulbactam mortality was 19.0% versus 32.3% of the colistin arm, treatment difference of −13.2%; 95% CI: −30.0, 3.5) [28].

In our systematic review, we included 16 studies dealing with the possible use of durlobactam in the treatment of MDR *A. baumannii* infections. The included studies were extremely heterogeneous, being studies performed in different settings with different designs.

Moreover, we could identify only one phase III clinical trial, providing data on the efficacy of durlobactam and sulbactam in the treatment of infections due to *A. baumannii*.

The results coming from the first studies evaluating the use of durlobactam against *A. baumannii* placed great hope in this novel beta-lactam inhibitor. The combination of durlobactam and sulbactam shows excellent in vitro potency against *A. baumannii* isolates, including MDR isolates that were resistant to carbapenems, aminoglycosides, tetracyclines and polymyxins.

Moreover, durlobactam pharmacokinetic and pharmacodynamic profiles suggest that it may be considered in the treatment of the most common *A. baumanni* infections, such as blood stream infections and pneumonia.

To consider, among the weaknesses of this novel compound, durlobactam does not inhibit class B beta-lactamases, i.e., NDM-1. This may halt durlobactam future usefulness, although fortunately the most recent surveillance studies report that infection of *A. baumannii* producing B metallo-beta-lactamases are globally rare [20].

Another factor that may halt the benefit of durlobactam/sulbactam use against infection due to *A. baumannii* is the potential development of resistance.

## 5. Conclusions

Certainly, data from randomized clinical trials will be required to confirm the effectiveness of durlobactam/sulbactam to treat infections caused by *A. baumannii*. In this regard, we are awaiting with interest the definitive results from the ongoing phase III open label clinical trial ATTACK (Acinetobacter Treatment Trial Against Colistin; NCT03894046). In the ATTACK trial, patients were randomized to receive either durlobactam/sulbactam plus imipenem/cilastatin or colistin plus imipenem/cilastatin. Probably, this therapy regimen is more reflective of real-world practice with MDR *A. baumannii* than monotherapy regimens employed in previous trials. However, this may lead to difficulty in interpreting the stand-alone efficacy of the durlobactam/sulbactam combination.

Positive results from this ongoing trial and from future clinical trials would enable again the possibility to treat infections caused by MDR *A. baumannii* with this unusual, dual beta-lactamase inhibitor combination.

## Figures and Tables

**Table 1:** Summary description of in vitro studies on durlobactam included in the systematic review.

Author & Country	Year	Study Aim		Study Design & Setting	Methods		Study Results
Durand-Réville TF et al. [13] US	2017	To modify the diazabicyclooctanone scaffold shared by traditional beta-lactamase inhibitors to extend its spectrum of activity to include a broad range of class D, A and C beta-lactamases		In vitro study Laboratory	Reverse phase chromatographies		Durlobactam was discovered. A novel broad-spectrum serine beta-lactamase inhibitor to be combined with a beta-lactam to treat patients infected by Gram-negative bacteria
Iyer R et al. [26] US	2018	To study the permeation characteristics of the *A. baumannii* outer membrane porin “OmpA” of substrates including durlobactam		In vitro study Laboratory	A specific whole-cell approach called titrable outer membrane permeability assay system was used to characterize the structure porin-permeation relationships. Antibacterial assays used a standard MIC format in Mueller-Hinton cation-adjusted broth. Contribution of OmpA to bacterial fitness was evaluated using a murine thigh model of infection		Durlobactam and sulbactam are substrates of OmpA, with a potential for structure-porin- permeation relationships
Shapiro AB et al. [15] US	2017	To assess the reversibility of durlobactam acylation of a set of beta-lactamases		In vitro study Laboratory	“Jump Dilution” to measure beta-lactamases off-rate constants; Mass spectrometry to assess durlobactam degradation		Durlobactam recyclized and dissociated intact from beta-lactamases AmpC, CTX-M-15, P99, SHV-5 and TEM-1
**Author &** **Country**	**Year**	**Study aim**	**Setting**	**Antimicrobial susceptibility testing**	**Bacterial** **isolates**	**Infection source**	**Study result and minimum inhibitory** **concentration**
McLeod SM et al. [14] US	2020	To report the in vitro activity of durlobactam/ sulbactam and comparator antibiotics against clinical isolates of *A. baumanni-* *calcoaceticus* complex	Isolates collected in 2016 and 2017 from 209 medical centers in 31 different countries	In-house broth microdilution panels	1722 *Acinetobacter baumannii- calcoaceticus* complex 1420 *A. baumannii* isolates	bloodstream (13.9%), intra- abdominal (3.8%), respiratory tract (61.2%), urinary tract (18.3%), skin and soft tissue (0.8%), other (2.0%)	Durlobactam/sulbactam had a MIC_50_/MIC_90_ of 1 and 4 mg/L, respectively, compared to a MIC_50_/MIC_90_ of 16/64 mg/L for sulbactam alone
McLeod SM et al. [20] US	2018	To determine spontaneous resistance to sulbactam in the presence of 4 mg/L durlobactam	Pharmaceutical industry laboratory	Susceptibility testing was performed in cation-adjusted Mueller–Hinton broth	4 *A. baumannii* clinical isolates	-	Durlobactam/sulbactam had MICs between 0.5 and 1 mg/L. The frequency of resistance to durlobactam/sulbactam (4xMIC) was lower than 9.0 × 10^10^
Seifert H et al. [21] US, Germany	2020	To evaluate the activity of durlobactam/sulbactam against global isolates of carbapenem- resistant *A. baumannii*	Isolates collected between 2012 and 2016 from 94 hospitals in 37 different countries	Broth microdilution	246 carbapenem-resistant *A. baumannii* isolates	The isolates were collected from various body sites	Durlobactam/sulbactam MIC_50_ and MIC_90_ values were 0.25 and 0.5 mg/L, respectively
Yang Q et al. [22] China	2020	To determine the in vitro activity of durlobactam/sulbactam against *A. baumannii* isolates	*A. baumannii* clinical isolates were collected from 22 sites across China between 2016 and 2018	Frozen microbroth dilution panels	982 *A. baumannii* clinical isolates. (831 (84.6%) were imipenem resistant)	Lower respiratory tract (715 isolates, 72.8%), intra- abdominal (170 isolates, 17.3%), urinary tract (59 isolates, 6.0%), skin and soft tissue (35 isolates, 3.6%) and blood (3 isolates, 0.3%)	Sulbactam/durlobactam was equally active against *A. baumannii* isolates from all infection types: the MIC_90_ was 2 mg/L for isolates from lower respiratory tract, intraabdominal and skin infections and 1 mg/L for urinary tract isolates. The MIC_90_ of sulbactam/durlobactam was 0.5 and 2 mg/L for carbapenem-susceptible and -resistant *A. baumannii* isolates, respectively
Nodari CS et al. [24] Brazil	2021	To evaluate the antimicrobial activity of durlobactam/sulbactam against a collection of MDR *A. baumannii* isolates	Isolates collected between 2000 and 2019 in Brazil	Broth microdilution method using durlobactam at a fixed concentration of 4 mg/L	112 MDR *A. baumannii* clinical isolates	-	Durlobactam/sulbactam MIC_90_ values of 4 mg/L
Barnes MD et al. [25] US	2019	To test the susceptibility of *A. baumannii* isolates to durlobactam/sulbactam and characterize the ability of durlobactam to inhibit class C and class D beta lactamases *Acinetobacter spp.*	Isolates collected in at four medical centers in US	Strains were phenotypically characterized using Mueller–Hinton agar dilution	98 *A. baumannii* clinical isolates. (43 were carbapenem resistant)	-	The MIC_90_ of the sole sulbactam was 32 mg/L, in comparison to a durlobactam/sulbactam MIC_90_ of 2 mg/L
Naha et al. [23] India	2021	To evaluate the efficacy of the durlobactam/sulbactam against clinical isolates of *A. baumannii*	Isolates collected between 2018 and 2019 in Indian hospital	Kirby–Bauer disc-diffusion method and broth micro-dilution. The efficacy of durlobactam was assessed through in silico intermolecular interaction analysis	28 *A. baumannii* clinical isolates	The 28 clinical strains were isolated from blood (n: 21) and sputum (n: 7)	93% of isolates expressed carbapenemases. Presence of carbapenemase genes resulted in sulbactam resistance (MIC: 16–256 mg/L) in all isolates. The intermolecular interactions of durlobactam and sulbactam with their respective targets displayed strong binding affinities against the strains of MDR *A. baumannii*

**Table 2:** Summary description of phase I, II and III clinical trials on durlobactam included in the systematic review.

Author, Year &Country	Study Population	Study Design	Study Aim	Setting	Methods	Study Results
Lickliter JD et al. [16] 2020 US, Australia	124	Randomized, double-blind, placebo- controlled phase I clinical trial	To evaluate the safety and pharmacokinetics of durlobactam, durlobactam/sulbactam and imipenem- cilastatin in healthy subjects	A single clinical site in Australia, between 2016 and 2017	4-part study. Part A was a single- ascending-dose escalation phase. Part B was a multiple-ascending-dose escalation phase. In parts C and D, the drug–drug interaction potential and the safety of durlobactam/sulbactam was investigated after single and multiple doses	On a total of 124 subjects, durlobactam was generally safe and well tolerated when it was administered either alone or in combination with sulbactam or imipenem–cilastatin. Renal excretion was the predominant clearance mechanism
Rodvold KA et al. [17] 2018 US	30	Phase I, multiple-dose open-label pharmacokinetic study in healthy adults	To determine and compare plasma, epithelial lining fluid and alveolar macrophage concentrations of durlobactam and sulbactam following intravenous administration	A single private facility in US, during 2017	Liquid chromatography-tandem mass spectrometry following repeated dosing of 1 g of durlobactam and 1 g of sulbactam every 6 h, for a total of 3 doses. A bronchoalveolar lavage was performed once in each subject	In 30 healthy adults subjects, durlobactam/sulbactam was safe and tolerated. Following the third infusion, durlobactam showed values of AUC 0–6 of 109.05 ± 23.44 mg/h/L, the half-life of 1.40 ± 0.18 h and a volume of distribution at steady state of 16.7 ± 3.0 L. Durlobactam AUC 0–6 based on mean epithelial lining fluid concentrations resulted 40.1 mg/h/L
O’Donnel J et al. [27] 2021 US	32	Placebo- controlled, single-infusion, phase I clinical trial	To evaluate the effect of a single supratherapeutic dose of durlobactam on the heart rate corrected QT interval in healthy volunteers	Private clinical pharmacology center	32 healthy volunteers were randomized to 1 of 6 sequences that included a single infusion of durlobactam 4 g, a single infusion of placebo, and a single infusion of placebo plus a single oral dose of moxifloxacin 400 mg given open-label at the end of the infusions. In each treatment period, Holter electrocardiogram measurements were obtained	No significant change was observed with durlobactam in comparison to placebo. A concentration-QT analysis demonstrated no significant effect of durlobactam on electrocardiogram parameters, including QT interval prolongation
O’Donnell J et al. [19] 2019 US	34	Phase I open-label, non-randomized study	To evaluate the effects of various degrees of renal impairment, including subjects with end-stage renal disease on hemodialysis, on the pharmacokinetics and tolerability of durlobactam and sulbactam	Three clinical sites in the United States between 2017 and 2018	Study included 8 patients with normal renal function, 26 patients with renal impairment. For healthy subjects and those with mild or moderate renal impairment, single 1 g dose each of durlobactam and sulbactam via 3 h infusion was administered, and for severe renal impairment, 500 mg doses were administered. For subjects on hemodialysis, 500 mg doses each of durlobactam and sulbactam were administered post-hemodialysis and pre-hemodialysis, with a 1-week washout between doses	Renal impairment had no effect of the safety profile of durlobactam and sulbactam. Decreasing renal function increased peak plasma concentration and AUC to durlobactam and sulbactam in a generally linear manner. Durlobactam exposure doubled in patients with renal impairment with creatinine clearance lower than 30 mL/min/1.73 m^2^. Hemodialysis was effective at removing both durlobactam and sulbactam from plasma
Sagan O et al. [18] 2020 Ukraine, Belarus, Bulgaria, Russia, US	80	Phase II double-blind, randomized, placebo- controlled trial	To evaluate the tolerability and pharmacokinetic of durlobactam/sulbactam in patients with complicated urinary tract infections	20 clinical sites in Belarus, Bulgaria, Russia, and Ukraine between January 2018 and May 2018	All the included patients received background therapy with imipenem, in addition to either durlobactam/sulbactam or placebo	The mean steady-state clearance and VD of durlobactam were 10.3 L/h and 31.6 liters, respectively. The microbiological intent-to-treat population were similar in the two groups, 36 (76.6%) and 17 (81.0%) patients in the durlobactam/sulbactam and in the placebo group, respectively
ATTACK trial [28] 2021 US	207	Open label, randomized phase III clinical trial	To evaluate the efficacy and safety of durlobactam/sulbactam in the treatment of patients with infections caused by *A. Baumannii-* *calcoaceticus* Complex	17 countries in the world, 95 clinical sites between 2019 and 2021	2-part study, with Part A being the randomized, controlled portion of the study in patients with *A. baumannii* hospital- acquired bacterial pneumonia or bacteremia. Part B is the single-group portion of the study and includes *A. baumannii* infections that are resistant to colistin. Part A contemplated two treatment arms: durlobactam/sulbactam (1 g/1 g qid) plus imipenem/cilastin (1 g/1 g qid) Versus colistin (2.5 mg/kg bid) plus imipenem/cilastin (1 g/1 g qid)	This clinical trial is ongoing. Recruitment phase ended with 207 participants. Durlobactam/sulbactam met the primary efficacy endpoint of 28-day all-cause mortality compared to colistin in the carbapenem- resistant *Acinetobacter* microbiologically modified intent-to-treat population (125 patients). Durlobactam/sulbactam mortality was 19.0% (12/63) compared to 32.3% (20/62) in the colistin arm (treatment difference of −13.2%; 95% CI: −30.0, 3.5).

AUC: area under the concentration-time curve; AUC 0–6: area under the concentration–time curve from 0 to 6 h; VD: volume of distribution.

## Supplementary Materials

The following supporting information can be downloaded at: https://www.mdpi.com/article/10.3390/jcm11123258/s1, Table S1: PRISMA abstract checklist; Table S2: PRISMA checklist.
